# A Single Application of Cerebellar Transcranial Direct Current Stimulation Fails to Enhance Motor Skill Acquisition in Parkinson’s Disease: A Pilot Study

**DOI:** 10.3390/biomedicines11082219

**Published:** 2023-08-08

**Authors:** Lidio Lima de Albuquerque, Milan Pantovic, Mitchell Clingo, Katherine Fischer, Sharon Jalene, Merrill Landers, Zoltan Mari, Brach Poston

**Affiliations:** 1 School of Health and Applied Human Sciences, University of North Carolina Wilmington, Wilmington, NC 28403, USA; limadeal@uncw.edu; 2 Department of Kinesiology and Nutrition Sciences, University of Nevada Las Vegas, Las Vegas, NV 89154, USA; milan.pantovic@unlv.edu (M.P.); fische74@unlv.nevada.edu (K.F.); sharon.jalene@unlv.edu (S.J.); 3 School of Medicine, University of Nevada Las Vegas, Las Vegas, NV 89154, USA; clingom@unlv.nevada.edu; 4 Department of Physical Therapy, University of Nevada Las Vegas, Las Vegas, NV 89154, USA; merrill.landers@unlv.edu; 5 Movement Disorders Program, Cleveland Clinic Lou Ruvo Center for Brain Health, Las Vegas, NV 89106, USA; mariz@ccf.org

**Keywords:** Parkinson’s disease, transcranial direct current stimulation, motor skill, cerebellum, cerebellar stimulation, motor learning, manual dexterity, dopamine, basal ganglia, transfer of motor learning

## Abstract

Parkinson’s disease (PD) is a progressive neurodegenerative disorder that leads to numerous impairments in motor function that compromise the ability to perform activities of daily living. Practical and effective adjunct therapies are needed to complement current treatment approaches in PD. Transcranial direct current stimulation applied to the cerebellum (c-tDCS) can increase motor skill in young and older adults. Because the cerebellum is involved in PD pathology, c-tDCS application during motor practice could potentially enhance motor skill in PD. The primary purpose was to examine the influence of c-tDCS on motor skill acquisition in a complex, visuomotor isometric precision grip task (PGT) in PD in the OFF-medication state. The secondary purpose was to determine the influence of c-tDCS on transfer of motor skill in PD. The study utilized a double-blind, SHAM-controlled, within-subjects design. A total of 16 participants completed a c-tDCS condition and a SHAM condition in two experimental sessions separated by a 7-day washout period. Each session involved practice of the PGT concurrent with either c-tDCS or SHAM. Additionally, motor transfer tasks were quantified before and after the practice and stimulation period. The force error in the PGT was not significantly different between the c-tDCS and SHAM conditions. Similarly, transfer task performance was not significantly different between the c-tDCS and SHAM conditions. These findings indicate that a single session of c-tDCS does not elicit acute improvements in motor skill acquisition or transfer in hand and arm tasks in PD while participants are off medications.

## 1. Introduction

Parkinson’s disease (PD) is the second most common neurodegenerative disorder and affects approximately one million people in the United States with annual costs approaching USD 11 billion [1]. The cardinal pathologic feature of PD is the loss of dopaminergic neurons in the substantia nigra pars compacta, which leads to striatal dopamine depletion. The decrease in dopamine is associated with a variety of motor deficits such as rigidity, bradykinesia, tremor, and postural instability that lead to severe impairments in the ability to perform daily living activities. Current surgical and pharmacological treatments may be affected by many problems including side effects, costs, and limited efficacy [2]. For example, Levodopa combined with other medications represents the standard treatment for PD, but its efficacy diminishes over time and leads to side effects such as dyskinesia [3]. Therefore, the development of practical and effective therapeutic adjuncts to complement current treatments remains an important priority in PD. 

Transcranial direct current stimulation (tDCS) is a non-invasive brain stimulation technique that can increase motor skill in healthy adults [4,5,6] (for review see [7]) and in PD [2,8] when delivered to the primary motor cortex (M1-tDCS). Although M1 has been the brain region most frequently targeted with tDCS, several studies have shown that the tDCS of the cerebellum (c-tDCS) can also enhance motor abilities [9]. For example, c-tDCS improved motor skill [10], motor learning [11], and performance in motor adaptation paradigms in young and older adults [12]. These studies could be relevant to intervention therapy development in PD because the cerebellum contributes to PD pathophysiology [13,14]. Specifically, the approach most often proposed [7] is to utilize the methodology developed from motor skill and learning studies involving tDCS to rehabilitation protocols used in clinical practice. The strategy of simultaneously combining tDCS with existing motor rehabilitation techniques would likely improve motor function to a greater extent than rehabilitation alone [7]. These effects have been shown to occur in a number of M1-tDCS studies and accumulating evidence has begun to demonstrate that c-tDCS could have similar or even greater effects [15] on motor skill compared to M1-tDCS in certain experimental conditions or motor tasks. Thus, c-tDCS could be a valuable and viable intervention for improving motor function in PD if it could enhance motor skill learning to a similar degree as seen in studies involving young and older adults.

Although PD is primarily a basal ganglia disorder, cerebellar involvement in PD pathophysiology provides a basis for targeting it with tDCS [13,14]. While M1 plays the predominant role in skilled execution of hand and arm movements [16], the descending drive of M1 to the spinal motor neuron pools of upper limb muscles depends on input from many motor areas onto intracortical interneurons [17] including crucial cerebellum contributions [17,18]. Furthermore, previously unknown bi-directional pathways have recently been discovered between the cerebellum and basal ganglia [19]. The cerebellar connections to basal ganglia and M1 are important because the effects of tDCS can extend to brain areas not stimulated directly. For example, M1-tDCS has been shown in animal, imaging, and pharmacological studies to induce remote effects in interconnected regions including the basal ganglia [20], thalamus [21], and the spinal cord [22]. Accordingly, M1-tDCS activation of basal ganglia in PD monkeys was associated with enhanced motor function [23]. This provides theoretical support that c-tDCS could indirectly impact M1 and basal ganglia activity. Cerebellar dysfunction in PD could also be a compensatory mechanism to diminish the negative effects of altered basal ganglia activity as people with PD with greater cerebellar activity exhibit better motor function [13]. Thus, c-tDCS could possibly augment motor skill in PD by heightening compensatory processes through increased cerebellar activation [24]. Finally, the increases in motor skill elicited by c-tDCS in older adults [12] are promising because evidence suggests that the cerebellum is the primary brain area responsible for movement impairments in older adults [25]. These factors along with the motor performance improvements elicited by c-tDCS in young adults [10,11,26] provide strong rationale for the investigation of c-tDCS to improve motor function in PD. 

Despite these lines of reasoning, only two studies [27,28] have examined the influence of c-tDCS on upper motor limb motor skill acquisition in PD, which is much fewer than the number of M1-tDCS investigations in PD [2,8]. Two additional c-tDCS studies have focused entirely on the lower extremities. One study only involved seven subjects [29] and found that only one of four different c-tDCS electrode montages improved balance performance. The other study found that c-tDCS did not improve dual task gait performance in PD [30]. Additionally, Ferrucci et al. (2016) reported that five days of c-tDCS did not improve any motor or cognitive rating scale measures except dyskinesia scores [31]. In the two aforementioned studies conducted in our laboratory, individuals with PD were only tested in the ON-medication state while c-tDCS was delivered [27,28]. Furthermore, these studies also had the limitation of being between-subjects experimental designs, which introduces numerous interindividual genetic and physiological differences that could potentially impact results [32,33]. Therefore, the primary purpose was to examine the influence of c-tDCS on motor performance in a complex, visuomotor isometric precision grip task (PGT) in PD in the OFF-medication state. The secondary purpose was to determine the influence of c-tDCS on the transfer of motor performance in PD as all but one of the previous c-tDCS studies in PD did not evaluate the transfer of motor skill learning [27,29,30,31]. It was hypothesized that c-tDCS would increase motor skill acquisition in the PGT compared to the SHAM stimulation. Finally, it was predicted that c-tDCS would increase motor skill in the transfer tasks to a greater extent than SHAM stimulation. 

## 2. Materials and Methods

### 2.1. Experimental Design

This pilot study utilized a double-blind, SHAM-controlled, within-subjects, counterbalanced design. The study was designed to determine the influence of c-tDCS on both motor skill acquisition and the transfer of motor skill. The assessment of the effect of c-tDCS on motor skill acquisition was accomplished by applying c-tDCS simultaneously with a task that was practiced extensively (PGT). In contrast, the transfer of motor skill measurement was accomplished by measuring performance in motor tasks involving many of the same hand and arm muscles as the PGT when c-tDCS was not applied concurrently and the tasks were not practiced extensively. The transfer tasks (see Section 2.6) included maximum voluntary contractions (MVCs), the Unified Parkinson’s Disease Rating Scale Part III (UPDRS-III), the Purdue Pegboard Test (PPT), and the Jebsen Taylor Hand Function Test (JTT). Overall, the rationale was that if c-tDCS could successfully enhance motor skill acquisition when applied during a motor task as well as elicit performance improvements in other hand and arm tasks, it would provide strong evidence that c-tDCS application could be translated to clinical settings and paired with practice and rehabilitation tasks.

### 2.2. Participants

Sixteen participants diagnosed with idiopathic PD (10 males, 6 females; mean age: 68.4 ± 11.8) participated in the study with 5, 9, and 2 participants being Hoehn and Yahr scale 1, 2, and 3, respectively. Thirteen participants were right-hand dominant and predominantly right-side affected, whereas three participants were left-hand dominant and predominantly left-side affected. The Montreal Cognitive Assessment (MoCA) was used to screen for early identification of cognitive impairment. Participants were required to have a MoCA score of 26 or higher to participate in the study (mean score 28.31 ± 1.70). Participants were free of other neurological disorders and did not meet international exclusion criteria for non-invasive brain stimulation studies [34]. The study was conducted according to the Declaration of Helsinki and approved by the Institutional Review Board at the University of Nevada Las Vegas.

### 2.3. Experimental Procedures

Two experimental sessions were performed and separated by a 7-day washout period. This within-subject, fully counterbalanced design was chosen for several reasons: (1) The substantial interindividual differences in the responsiveness to tDCS due to physiological, biological, and anatomical factors are mitigated [32,33] with within-subjects designs. For the cerebellum in particular, there are variations in nerve fiber orientation and convolution of the cerebellar cortex beneath where the tDCS electrodes are placed [35]. (2) The within-subjects design allowed for greater statistical power compared with a between-subjects design [36] such as that employed in previous c-tDCS and PD studies performed in our laboratory [27,28]. (3) Many prior M1-tDCS studies in PD [8] and a previous c-tDCS study in PD [29] have had success with within-subject designs using similar motor tasks and washout periods, despite having fewer participants than the current study. 

**Figure 1:**
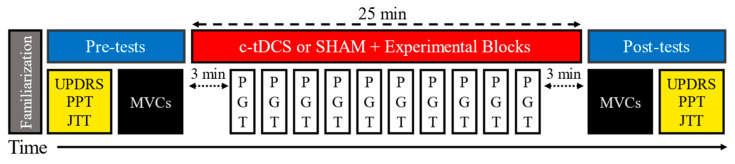
Schematic representation of the experimental protocol that comprised a familiarization, pre-tests (UPDRS-III, PPT, JTT, MVCs), 25 min of either c-tDCS or SHAM stimulation concurrent with 10 trials of the PGT, and post-tests (MVCs, UPDRS, PPT, JTT) in the order depicted.

In both experimental sessions, participants reported to the lab in the morning after a 12 h medication withdrawal. This corresponds to the practically defined OFF-medication condition [37], which standardizes clinical responses. All patients kept their medication schedule constant throughout the study period. Participants were tested in the OFF-medication state so that the influence of c-tDCS on motor performance in the basic disease state could be determined and because the aforementioned previous c-tDCS studies in PD from our laboratory were performed in the ON-medication state in a between-subjects design [27,28]. The PGT was performed over a 25 min period concurrent with either c-tDCS or SHAM stimulation with the predominately affected hand. Additionally, the transfer tasks (MVCs, UPDRS-III, PPT, and JTT) were completed before and after stimulation (pre- and post-tests; see below) and were all performed with the predominately affected hand. These tasks were considered transfer tasks because they were not conducted simultaneously with c-tDCS and were not practiced. Accordingly, each experiment was performed in the order prescribed: (1) a familiarization that included acquainting participants with the motor tasks along with visual demonstrations by the investigators; (2) pre-tests were completed; (3) c-tDCS or SHAM stimulation was applied and 10 trials of the PGT were performed during the stimulation period; and (4) post-tests were completed (Figure 1).

### 2.4. Pinch Grip Task (PGT)

The PGT arrangement was similar to previous studies [27,38]. Briefly, participants were seated with the dominant arm abducted to 45°, the elbow flexed to 90°, and the hand semi-supinated while resting on a table. The PGT involved accurately matching a target sine wave (0.5 Hz) on a monitor by producing isometric force using a precision grip (index finger and thumb) against a grip device instrumented with force transducers. The sine wave minimums and maximums were 5% and 25% of the pre-test MVC force for all PGT trials. Thus, the average force produced during each trial was approximately 15% of MVC, but the participants had to modulate this force between 5% and 25% of MVC by accurate force production and force relaxation within this range. Each PGT trial involved matching the template for 30 s and a total of 10 trials were performed concurrent with either c-tDCS or SHAM. The stimulator was turned on for three minutes prior to performing the first PGT trial and was kept on (~1–3 min) after the last trial [10]. 

The PGT was the motor task chosen to be paired with stimulation for several interrelated reasons: (1) Numerous M1-tDCS studies [7,39,40] and a c-tDCS study in young adults [11] have found that tDCS elicits large, acute performance increases in precision grip tasks. Thus, the PGT had potential for improvement due to c-tDCS. (2) fMRI has revealed that the cerebellum participates in force amplitude and rate modulation in the precision grip [41]. (3) The precision grip is a functional multi-muscle task required in everyday living. (4) There is high cerebellar involvement in muscle activation timing, error detection [42] in voluntary movements, and in visuomotor tracking [43], which are all motor control aspects of the PGT. (5) A series of behavioral and fMRI studies in PD by Vaillancourt and colleagues have shown that the basal ganglia is highly involved in various aspects of pinch grip task performance (e.g., amplitude modulation, rate of force production, force relaxation) and displays differences compared to healthy controls and other disease states [44,45,46,47,48,49,50,51]. 

### 2.5. Cerebellar Transcranial Direct Current Stimulation (c-tDCS)

A NeuroConn DC Stimulator delivered anodal tDCS via two rubber electrodes (5 × 5 cm) encased in saline-soaked sponges. c-tDCS was applied over the cerebellum ipsilateral to the predominantly affected hand (anode 3 cm lateral to the inion, cathode over the ipsilateral buccinator muscle, current strength 2 mA, 25 min duration). These c-tDCS parameters have elicited large, immediate motor performance increases in young and older adults [10,11,12,26]. For SHAM, the current was ramped up and down over 30 s according to standard SHAM procedures. The stimulator was programmed by an investigator who did not participate in data collection and the investigators who conducted the experiments were blind to the experimental conditions as in our previous studies [10,27,28,52]. 

### 2.6. Transfer Tasks

Four tasks were employed to quantify transfer of motor performance to tasks that were not performed during stimulation or practiced as extensively as the PGT. The transfer tasks (MVCs, UPDRS, PPT, and JTT) were administered immediately before and after the stimulation and practice period on the predominantly affected hand/arm. Execution of the tasks after stimulation was an important study design aspect because numerous studies have shown that tDCS can elicit performance enhancements for at least 30 min after stimulation. Accordingly, the transfer tasks were able to be completed in this 30 min time period after the application of c-tDCS had ended.

Three MVC trials were completed in the identical experimental arrangement and hand posture as in the PGT. Similar to previous studies, participants exerted maximum force in the shortest time possible and held the maximum for 5 s [53]. The MVC served three purposes: (1) the pre-test MVC force was used as a reference value to calculate the PGT target forces; (2) the difference between the pre- and post-test MVCs was used to rule out the influence of muscle fatigue on the PGT results; and (3) MVC served as a transfer task representing the motor ability of maximum strength as a recent study in young adults found that c-tDCS could acutely enhance maximal isometric force production in a full-body task [54]. The UPDRS-III was administered by an investigator trained by a movement disorder neurologist. The UPDRS-III was chosen as a transfer task because it is the gold standard clinical test to rate motor symptoms in PD. The PPT is a standard test to evaluate arm and hand function and entails picking up and placing pegs in small holes as fast as possible over 30 s. Similarly, the JTT is a manual dexterity assessment used in aging, movement disorder, and tDCS studies. Six tasks are performed that imitate common tasks of daily living as quickly as possible with the hand and arm. The PPT and JTT were each performed for three trials. The PPT and JTT were selected as transfer tasks as they are among the most common manual dexterity tests utilized in movement disorder research and tDCS studies. Collectively, these transfer tasks were chosen because they are well-characterized in the literature, provide information on several aspects of motor function, have varying emphasis on proximal and distal upper limb muscle control, and exhibit varying degrees of overlap of the muscle groups involved in the PGT. 

### 2.7. Data Analysis 

The force error in the PGT was the primary dependent variable, whereas MVC, UPDRS-III, PPT, and JTT were secondary dependent variables. Force error in the PGT was calculated as the average error in force relative to the target over each 30 s trial [27]. Specifically, the force error was calculated in the following steps: (1) the difference in the target force displayed on the template and the force produced was quantified at each sampling point for the 30 s trial; (2) the absolute value of each of these differences was calculated; (3) the average of all of these absolute values was quantified for the entire trial; and (4) the final force error value was taken as the average of the 10 PGT trials (grand average). MVC force was quantified as the average force produced during the plateau period for each trial and the highest force among the 3 trials was denoted as the MVC. The UPDRS score was quantified as the sum of the items on the motor examination Part III. The PPT score was calculated as the average number of pegs over 3 trials. For the JTT, the total time to complete the 6 tasks was computed for each trial and averaged over 3 trials. 

### 2.8. Statistical Analysis

Force error in the PGT between the c-tDCS and SHAM conditions was compared with a two-tailed paired *t*-test, whereas MVC, UPDRS, PPT, and JTT were analyzed with two-way (2 *condition* (c-tDCS, SHAM) × 2 *test* (pre, post)) within-subjects ANOVAs. The significance level was α = 0.05 and data are indicated as means ± standard errors in the figures. Similar to our previous study [28], an interim futility analysis was conducted after the completion of 16 participants to estimate the sample size needed for the primary outcome variables (PGT, UPDRS-III, MVC, Pegboard, JTT). 

## 3. Results

### 3.1. PGT

**Figure 2:**
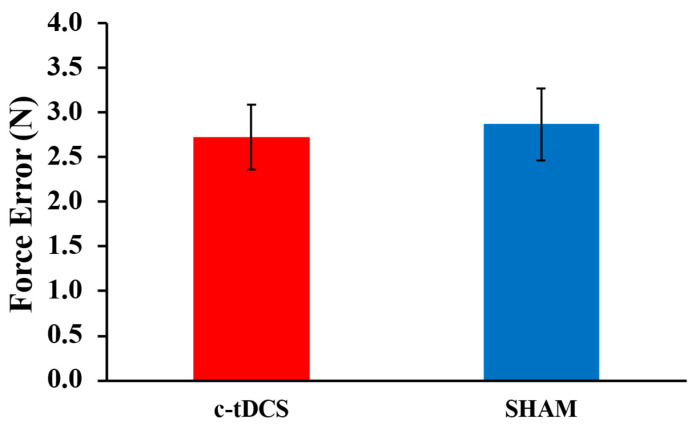
Force error in the PGT for the c-tDCS and SHAM conditions. The force error was not significantly different between the c-tDCS and SHAM conditions (*p* = 0.322).

The force error was not significantly different between the c-tDCS and SHAM conditions (Figure 2; Table 1).

### 3.2. Transfer Tasks

**Figure 3:**
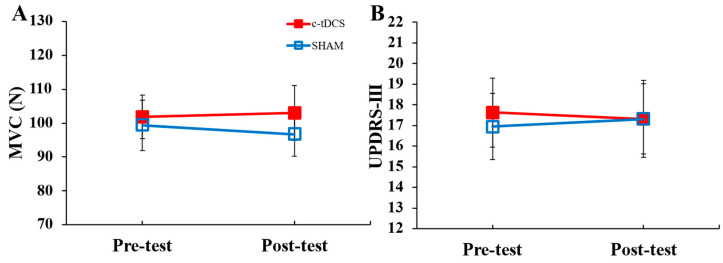
MVC forces and UPDRS-III scores in the pre- and post-tests for the c-tDCS (closed squares) and SHAM conditions (open squares). (**A**) MVC forces were not significantly different for the pre- and post-tests (*p* = 0.749) or between the c-tDCS and SHAM conditions (*p* = 0.114). (**B**) UPDRS scores were not significantly different for the pre- and post-tests (*p* = 0.920) or between the c-tDCS and SHAM conditions (*p* = 0.709).

**Figure 4:**
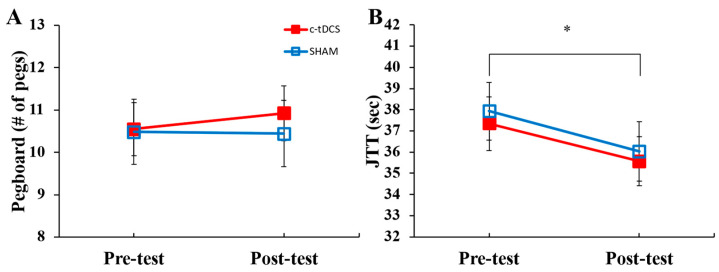
PPT scores and JTT times in the pre- and post-tests for the c-tDCS (closed squares) and SHAM conditions (open squares). (**A**) PPT scores were not significantly different for the pre- and post-tests (*p* = 0.268) or between the c-tDCS and SHAM conditions (*p* = 0.412). (**B**) JTT times were significantly lower in the post-test compared to the pre-test (*p* = 0.004) but not between the c-tDCS and SHAM conditions (*p* = 0.607). * Indicates a significant difference between the pre- and post-tests.

For the MVC, the main effect for *condition*, main effect for *test*, and *condition* × *test* interaction were all non-significant (Figure 3A; Table 1). Similarly, the main effect for *condition*, main effect for *test*, and *condition* × *test* interaction were all non-significant for the UPDRS-III (Figure 3B; Table 1). For the PPT, the main effect for *condition*, main effect for *test*, and *condition* × *test* interaction were all non-significant (Figure 4A; Table 1). For the JTT, the main effect for *condition* and *condition* × *test* interaction were non-significant (Figure 4B; Table 1). However, there was a significant main effect for *test*, which indicated that JTT time was shorter in the post-test compared to the pre-test (Figure 4B; Table 1). 

### 3.3. Futility Analyses

Interim futility analyses were conducted on all the dependent variables to determine if additional participant recruitment was needed. Using the means, standard deviations, and test statistics from these analyses and the “Conditional Power and Sample Size Reestimation of Paired T-Tests” and the “Conditional Power and Sample Size Reestimation of Tests for Two Means in a 2 × 2 Cross-Over Design” modules on PASS 20.0.10 (NCSS, LLC, Kaysville, UT, USA), it was determined that the following numbers of participants were needed to achieve sufficient power to find statistically significant differences: 115 (PGT), 125 (UPDRS-III), 220 (MVC), 73 (Pegboard), and 4676 (JTT). Based on these estimates and the impracticality of recruiting these numbers, it was decided to terminate recruitment of additional participants for futility because of the lack of meaningful treatment effects.

In summary, the statistical results clearly indicated that c-tDCS failed to elicit significant increases in motor skill acquisition or transfer compared to SHAM stimulation. In fact, the mean changes in all of the dependent variables were almost identical between the c-tDCS and SHAM conditions and no trends for any c-tDCS effects emerged. Accordingly, the futility analysis indicated that it was highly unlikely that the lack of significant differences between the c-tDCS and SHAM conditions was due to the sample size of the current study.

## 4. Discussion

Parkinson’s disease (PD) is a progressive neurodegenerative disorder that leads to numerous impairments in motor function that compromise the ability to perform activities of daily living. The current pharmaceutical, surgical, and management strategies for PD are directed towards relieving the symptoms associated with the disease. Levodopa combined with other medications represents the standard treatment for PD, but its efficacy diminishes over time and leads to side effects such as dyskinesia. Accordingly, practical and effective adjunct therapies are needed to complement current treatment approaches in PD. Non-invasive brain stimulation techniques such as M1-tDCS and c-tDCS have emerged as potential valuable adjunct interventions with a realistic potential to be translated into clinical practice to improve motor performance in PD. Based on the available motor skill and rehabilitation studies to date, the most common strategy proposed to realize this goal has been to concurrently apply tDCS with existing motor rehabilitation techniques, which should improve function to a greater degree than rehabilitation alone.

Therefore, the primary purpose was to examine the influence of c-tDCS on motor skill acquisition in a complex, visuomotor isometric precision grip task (PGT) in PD, whereas the secondary purpose was to determine the influence of c-tDCS on the transfer of motor skill in PD. This was accomplished by delivering c-tDCS simultaneously with a motor task (PGT) during practice to measure skill acquisition. The transfer of motor skill measurement was achieved by measuring performance before and after c-tDCS in motor tasks involving the same hand and arm muscles as the PGT. The study produced two main findings. First, c-tDCS did not significantly improve motor performance in the PGT task relative to SHAM. Second, transfer task performance was not enhanced following c-tDCS application relative to SHAM. Taken together, the results indicate that a single session of c-tDCS does not elicit improvements in motor skill acquisition or transfer of motor skill in hand and arm tasks in PD in the OFF-medication state. 

The current study sought to extend previous studies that found that c-tDCS applied concurrently with motor task execution could enhance motor skill acquisition and motor learning in young and older adults [9,10,11,12]. Based on these observations and the cerebellar involvement in PD, the study investigated the effects of c-tDCS on motor skill acquisition and transfer in PD. It was originally hypothesized that force error in the PGT would be lower during c-tDCS compared with SHAM stimulation. Contrary to this prediction, force error in the PGT during c-tDCS application was only ~5% lower in the c-tDCS condition, but this small difference did not approach statistical significance (*p* = 0.322). This is in contrast to most previous c-tDCS studies in young and old adults, including a study from our laboratory [10] where c-tDCS increased throwing accuracy in young adults. The results are also not consistent with the motor function improvements observed in the majority of M1-tDCS studies in PD [2,8]. However, a study in young adults reported that c-tDCS failed to improve performance in a whole-body dynamic balance task [55]. Additionally, a single session of c-tDCS did not enhance clinical writing task scores of people with dystonia [56]. This is relevant because, similar to PD, dystonia is primarily a basal ganglia disorder that is also characterized by cerebellar contributions to impaired movement. Accordingly, a previous study from our laboratory involving a between-subjects design by Albuquerque et al. (2020) [27] found that a single application of c-tDCS did not enhance motor skill learning in PD while participants were in the ON-medication state, which confirms and extends the current findings. Overall, it appears that c-tDCS may not be effective, at least in acute conditions for upper limb tasks, at improving motor function in the movement disorders such as dystonia and PD where cerebellar deficits play a partial role.

A critical issue in tDCS studies and the field of motor control is whether performance improvements realized in a given task can be generalized (transferred) to other motor tasks [57]. Any modality will have limited utility if effects are only elicited when given simultaneously with a particular motor task, because it would be impractical for people with PD to train every impaired task of daily living. Surprisingly, the effects of tDCS on motor transfer have only been investigated in a few studies in any population and have yielded conflicting results [58,59,60]. In the present study, c-tDCS failed to improve performance of any of the transfer tasks as indicated by the lack of change between the pre-tests and the post-tests. These findings are in contrast to an M1-tDCS study in young adults, which demonstrated that tDCS increased transfer in some but not all aspects of arm movement performance [59]. In contrast, the results are consistent with an older adult study where improvements in the trained task conducted simultaneously with M1-tDCS did not generalize to other hand tasks [60]. Furthermore, M1-tDCS-induced improvements in a pinch grip task did not transfer to PPT or JTT performance in people who have had a stroke [58], which is particularly noteworthy as these motor tasks were almost the exact same as the motor tasks employed in the present study. Overall, these findings suggest that M1-tDCS and c-tDCS may have limited ability to induce the transfer of motor performance in several populations including PD.

The dissimilar findings between the present study and the preponderance of M1-tDCS studies in PD and c-tDCS studies in young and older adults imply that it should not be presumed that c-tDCS always enhances motor abilities. Most importantly, the outcomes suggest that results in healthy populations do not always translate directly to PD. Although the current results were unexpected, there are several possible factors responsible for the failure of c-tDCS to improve motor performance. 

One possible explanation is that the balance of excitatory and inhibitory pathways from the cerebellum to M1 and basal ganglia are so altered in PD compared to healthy adults that c-tDCS application does not induce the same net motor effects. More specifically, the impairments in motor function in PD are mainly considered to be due to the loss of dopaminergic cells in the substantia nigra pars compacta that project to the striatum [3]. This results in lower levels of dopamine input onto the medium spiny neurons of the striatum that also receive cortical inputs and form part of the direct and indirect pathways which ultimately project back to the cortex. However, the motor dysfunctions in PD do not result solely from striatal dopamine depletion but are also due to impairments in the motor loops between the cerebellum and the cortex [3,13,14]. In healthy adults, transcranial magnetic stimulation (TMS) or tDCS of the cerebellum activates a cerebellar–thalamic–cortical pathway that bifurcates such that one pathway elicits a net inhibition on corticospinal output cells in M1, whereas the second pathway elicits a net facilitation [17]. In addition, two other TMS studies found that two interneuronal networks in M1 differ in how they process cerebellar inputs [61,62]. These studies also demonstrated that these pathways change during motor skill learning, which underscores their functional relevance [18]. Furthermore, similar effects could occur if there are imbalances in the bi-directional pathways between the cerebellum and basal ganglia [19]. Taken together, the widespread network dysfunction in numerous neural circuits between the cerebellum, basal ganglia, and M1 in PD might have led to a lack of positive c-tDCS effects on motor performance. 

A second possibility is that one acute c-tDCS application may be insufficient to increase motor function and multi-day stimulation (3–5 sessions) might be necessary. Consistent with this line of reasoning, it was argued in the aforementioned negative c-tDCS study in dystonia that one c-tDCS session is unlikely to be sufficient to override negative motor adaptations that have developed due to the disease over many years [40]. This proposal is supported by the fact that M1-tDCS studies [39,40,58] and a c-tDCS study [11] reported cumulative motor learning effects over 3–5 days of practice performed concurrent with stimulation. Nonetheless, in these studies, the effects of tDCS on performance also reached significance within the first day. Furthermore, the vast majority of M1-tDCS studies in healthy adults and in PD have been single-session studies and have reported significant effects [2,8]. Thus, it is not mandatory that repeated daily application of c-tDCS is necessary to detect enhancements in motor function in PD if they occur. Accordingly, a recent study from our laboratory found that 9 days of c-tDCS application over a 2-week period did not improve motor learning or transfer of motor learning to a greater extent than practice alone (SHAM stimulation) in PD [28]. That study differed from the current study in that testing was performed in both ON- and OFF-medication states. However, the lack of an effect of c-tDCS even though nine stimulation sessions were performed supports the single-session motor performance results of the current study. Specifically, nine stimulation sessions should have been more than enough time for c-tDCS effects to appear if present based on numerous 3–5 day M1-tDCS and c-tDCS studies [11,39,40,58].

It could also be argued that the c-tDCS parameters (montage, current, and duration) were not optimal as other studies in various other populations have had at least some success with slightly different parameters [9]. Although this is possible, it seems highly unlikely as identical parameters were successful in our previous study [6] and in other studies from a different research group [11,12,26,63,64] in young and older adults. These same c-tDCS parameters were selected for the present study and a previous c-tDCS study in PD [27] because they had elicited positive effects in the greatest number of studies and the magnitude of the performance improvements was quite high. Therefore, they were deemed the most likely c-tDCS parameters to initially utilize as no c-tDCS studies existed at the time in PD. However, it cannot be ruled out that other c-tDCS parameters may be more effective in PD. Recently, Workman et al. (2020) [29] compared the ability of four different c-tDCS montage/current strength paradigms (unilateral and bilateral; 2 mA and 4 mA) to improve gait and balance in PD. The findings indicated that only the bilateral electrode montage with a current strength of 4 mA improved balance performance in PD, whereas gait performance was not enhanced in any of the four c-tDCS conditions relative to SHAM. The use of a current strength of 4 mA was especially novel as only a handful of the hundreds of tDCS studies available in any population have employed such a high stimulation intensity. However, this study only involved seven participants and did not report if the testing was conducted in the ON- or OFF-medication state. Despite these issues and the fact that lower extremity function was tested as opposed to fine motor performance in the current study, the results of Workman et al. (2020) [29] demonstrate that many other combinations of c-tDCS parameters are possible and could potentially induce positive effects on motor skill in PD. Similarly, it could be argued that the c-tDCS montages employed have had more overall variability across studies compared with M1-tDCS montage arrangements. Although some M1-tDCS studies have employed bi-hemispheric montages or electrode arrangements where the reference electrode is on the shoulder, the vast majority have used the standard M1-supraorbital montage. In contrast, c-tDCS studies appear to have had more relative variability in montage parameters such as unilateral versus bilateral and the location of the reference electrode (e.g., shoulder versus buccinator muscle). Furthermore, some computational modeling research [65] has indicated that the most common and effective c-tDCS electrode montage, which was also used in the present study, can lead to a dispersion of current so that some current spills over to the contralateral cerebellar hemisphere. This effect was present in all age groups but was greater at ages 75 and above and was due to cerebellar shrinkage with age. Therefore, this phenomenon could have influenced the results of the present study due to the age of the participants. However, this c-tDCS montage has still elicited motor performance improvements in old adults [12], despite these possible effects. Taken together, these issues highlight the need for future research to investigate other promising c-tDCS stimulation protocols other than the one used in the present study and point to the possibility that the stimulation parameters of c-tDCS may need to be individualized for optimal results. Finally, the negative results on motor performance in the present study could be due to some combination of all the aforementioned factors above.

The study was subject to several limitations that should be acknowledged. First, it could be argued that the study had a relatively small sample size that did not allow the identification of performance improvements due to c-tDCS. Accordingly, the low sample sizes that have been used in neuroscience research in general have been recognized and can lead to problems involving reproducibility and incorrect conclusions [66,67]. However, the results of our futility analyses clearly indicated that the effect sizes were very small and there were no apparent treatment effects. Furthermore, the sample size of 16 in the current study was actually larger than the average tDCS motor skill study in healthy adults and in PD. Specifically, a close examination of an extensive tDCS motor skill study review [7] in healthy adults reveals that the average sample size per group was approximately 13 (see their tables 1–3), with 75–80% of these studies showing positive tDCS effects. Similarly, a review article and associated table of upper limb studies in PD [8] appears to show that the average sample size per group was approximately 11. Nonetheless, the current results need to be replicated in larger studies using appropriate sample size estimation to observe clinically relevant effects. 

A second limitation is that more than one c-tDCS session would be needed to be able to elicit statistically significant motor skill augmentations. This is a typical argument made in tDCS research in healthy adults and especially in motor disorder studies [56] where it is argued that one session cannot overcome years of disease-induced deficits in motor function. In addition, the fact that a series of M1-tDCS studies in healthy adults [39,40] and a c-tDCS study [11] found large cumulative effects over 3–5 days of stimulation supports this view. However, several lines of evidence argue against the lack of multiple sessions as a major reason for single-session tDCS studies failing to show positive performance effects: (1) all of the aforementioned multiple-day studies also reported significant effects within the first day; (2) the vast majority of acute studies in both healthy adults and in PD have shown positive effects (for reviews, see [2,7,8]); (3) a study in our lab that involved 9 days of c-tDCS in PD found no improvements in motor learning [28]; and (4) a recent meta-analytical review [68] found that tDCS efficacy was not affected by the number of stimulation sessions in healthy older adults or PD. Thus, it appears that while multiple tDCS sessions are highly desirable, they are not obligatory to demonstrate positive tDCS effects if they exist. 

A final limitation was that the study did not have an age-matched healthy control group consisting of older adults, which would have allowed a direct comparison between groups in the same experimental and laboratory conditions. If the control group would show improvement, this would be stronger evidence that c-tDCS can be effective in older adults but not in PD due to their widespread basal ganglia, cortical, and cerebellar dysfunction. Thus, the current results can only be compared to prior studies by other research groups that have shown that c-tDCS can improve motor skill in healthy older adults [12,69]. In summary, the results of the present study should be interpreted with caution based on the aforementioned limitations and more work is warranted to address these limitations in future research.

In summary, one session of c-tDCS did not enhance motor skill or transfer of motor skill acquisition in hand and arm tasks in PD in the OFF-medication state. Taken together, this study provides evidence that the c-tDCS applied using the parameters that have enhanced motor skill in young [10,11,63,64] and old adults [12] does not elicit the same motor performance benefits in PD. There are several avenues for future c-tDCS research in PD. For example, some evidence has shown that c-tDCS can modulate some aspects of cognition in PD [70]. Another opportunity for future research is the use of tACS applied to the cerebellum as opposed to tDCS. Accordingly, tACS applied to the cerebellum [71] as well as to M1 and the cerebellum concurrently [72,73,74] has been shown to enhance motor function in other populations, but no cerebellar tACS studies in PD have been conducted to date. In addition, future c-tDCS studies should examine multiple stimulation sessions and different parameters of stimulation to fully determine the viability of c-tDCS as an intervention to improve motor function in PD. Specifically, individualized placement of tDCS electrodes using anatomical data from MRI and higher current strengths [29] than typically used could overcome the recently described interindividual differences in cerebellar anatomy that influence the amount of current reaching cerebellar neurons [35]. Future research in all of these areas will be needed to determine the viability of c-tDCS as a modality to improve motor function in PD and to utilize it in clinical settings.

## Figures and Tables

**Table 1:** Summary of statistical values. The corresponding statistical test, *p* values, and partial eta squared values for each of the dependent variables described in the text and figures above.

Dependent Variable	Statistical Test		*p*	η_p_^2^
PGT (N)	Paired *t*-test		0.322	
MVC (N)	2 × 2 within-subjects ANOVA	*condition*	0.224	0.158
		*test*	0.749	0.007
		*condition* × *test*	0.446	0.036
UPDRS-III (Score)	2 × 2 within-subjects ANOVA	*condition*	0.709	0.010
		*test*	0.920	0.001
		*condition* × *test*	0.341	0.061
Purdue Pegboard (pegs)	2 × 2 within-subjects ANOVA	*condition*	0.412	0.045
		*test*	0.268	0.081
		*condition* × *test*	0.222	0.098
JTT (sec)	2 × 2 within-subjects ANOVA	*condition*	0.607	0.018
		*test*	0.004	0.427
		*condition* × *test*	0.872	0.002

## Data Availability

The data presented in this study are available on request from the corresponding author.
